# Understanding and Overcoming COVID-19 Vaccine Hesitancy & Anti-Vaccine Beliefs Within the High Secure Forensic Services

**DOI:** 10.1192/bjo.2022.389

**Published:** 2022-06-20

**Authors:** Karla Giles, Anju Soni, Marc Head, William Gewanter, Christina Kalovidouri, Tomasz Tyszuk-smith

**Affiliations:** 1 College of North West London, London, United Kingdom; 2 Broadmoor Hospital, London, United Kingdom

## Abstract

**Aims:**

This is a cross-sectional service evaluation study of the vaccination programme within the high secure setting of Broadmoor hospital with a view of improving the quality of it's delivery. We aimed to establish patients views about COVID-19 vaccinations particularly if there are any themes as to why the patients choose/did not choose to receive the vaccine. This information will be used to help us understand how to overcome vaccine hesitancy and anti-vaccine beliefs.

**Methods:**

Patients across Eight wards were asked to participate in the study. 56 patients agreed to be administered the following semi-structured questionnaire by the doctors.

1. Have you had a COVID-19 vaccine?
2. Do you think there any advantages to taking a COVID-19 vaccine? Yes/No. If you think there are any advantages, please write these
3. Do you have any fears or worries about the COVID-19 vaccine? Yes/No. If you do have any fears or worries, please write these.

The results of this were reviewed and put into the categories that are cited below.

**Results:**

14 patients had no vaccination, 2 had one, 38 had two or more.

34 patients said there were advantages, 13 said no advantages and 9 did not know. The themes of the advantages were established: Protects you from bad infection and symptoms (48), stops you from passing it on to others (3), blank (13), others (13) which included “Important to follow government guidelines, proven through history to work, it was offered, I'm more concerned with hepatitis, The doctor would have my best interests.”

30 patients stated that they did have fears and 26 did not. Common themes established were; side effects (17), Not tested correctly/given too quickly (5), Blood clots (2), positive comments (2), blank (22), others (10), which included, “Interaction with medications, more fear about face masks, injecting humanity with something could kill them, infertile generation, Control the public, don't like injections and alter the DNA genome.” The common side effects of concern were “painful arm, fever and headache.”

**Conclusion:**

68% of patients had 2 or more vaccinations across the 8 wards studied. The commonest advantages cited by 86% of patients was to protect themselves from serious illness. The commonest fears or worries were of side-effects that result from the vaccine, although 46% patients had no worries and 39% gave no explanation for fears or worries. The fears and worries appeared mainly related to vaccine hesitancy rather than fixed generalised anti- vaccine views.

